# Validation of newly synthesized sex-determining PCR using immature *Ixodes scapularis* (Acari: Ixodidae) ticks

**DOI:** 10.1093/jme/tjaf138

**Published:** 2025-10-08

**Authors:** Cody W Koloski, Arvind Sharma, Benjamin Faustino, Nathan King, Monika Gulia-Nuss

**Affiliations:** Department of Veterinary Microbiology, Western College of Veterinary Medicine, University of Saskatchewan, Saskatoon, Canada; Department of Biochemistry and Molecular Biology, University of Nevada, Reno, USA; Department of Biochemistry and Molecular Biology, University of Nevada, Reno, USA; Department of Biochemistry and Molecular Biology, University of Nevada, Reno, USA; Department of Biochemistry and Molecular Biology, University of Nevada, Reno, USA

**Keywords:** ticks, male, female, sex determination

## Abstract

The ability to determine the sex of tick nymphs has the potential to answer questions concerning tick biology and pathogen ecology. A duplex polymerase chain reaction (PCR) that was recently developed for sex determination in adult *Ixodes scapularis* Say ticks was used to test whether the method could be applied to determine the sex of unfed *I. scapularis* larvae and nymphs. We found that the duplex PCR could be used to determine the sex of immature ticks and could also be used on small tissue segments from live nymphs.

## Introduction

Ticks pose a substantial health burden to both humans and animals. *Ixodes scapularis* in North America is an important vector for the spirochete pathogen, *Borrelia burgdorferi sensu stricto*, the causative agent of Lyme disease. In North America alone, hundreds of thousands of Lyme disease cases are reported annually, with an estimated economic burden of $3,000 USD per case (Ogden et al. 2004, Kugeler et al. 2021). The maintenance of *B. burgdorferi* in nature depends primarily on the immature tick stages (larvae and nymphs), both of which usually obtain blood meals from the same reservoir hosts (Piesman and Gern 2004). Understanding the transmission dynamics of *B. burgdorferi* during these immature stages is critical to uncovering patterns in pathogen population structure and spread.

One factor that is poorly characterized is the role that male and female immature ticks contribute to pathogen transmission. A key barrier to investigating this is the lack of visible sexual dimorphism in immature *I. scapularis* ticks. Current methods to determine sex at these stages often require sensitive equipment, technical expertise, or are only possible after rearing ticks to adulthood. For example, researchers have used sensitive scales to weigh replete nymphs, showing that females typically ingest larger blood meals and thus weigh more (Dusbabek 1996, Hu and Rowley 2000). However, replete weights can vary and are not always reliable indicators of sex. Subtle size differences between male and female nymphs also exist but require extensive training and time to detect (Dusbabek 1996).

Recently, a duplex polymerase chain reaction (PCR) method was developed to differentiate male and female *I. scapularis* adults and has already been applied to investigate sex-specific effects in *B. burgdorferi* infected ticks (Koloski et al. 2025, Ronai et al. 2025). This assay targets both an autosomal gene (present in both sexes) and a male-specific sequence, allowing for reliable sex identification and serving as an internal control for DNA quality.

While this PCR assay represents the first molecular tool for *I. scapularis* sex differentiation, it has only been validated on adult ticks. Its potential application to immature ticks could significantly benefit studies in tick-borne pathogen ecology. The objective of this short report is to extend the validation of this duplex PCR method to immature *I. scapularis*. To do so, we tested DNA from whole unfed larvae and nymphs, as well as from leg segments of unfed nymphs that were subsequently allowed to feed and molt into adults for sex confirmation.

## Materials and Methods

### Experimental Design

**Fig. 1.**
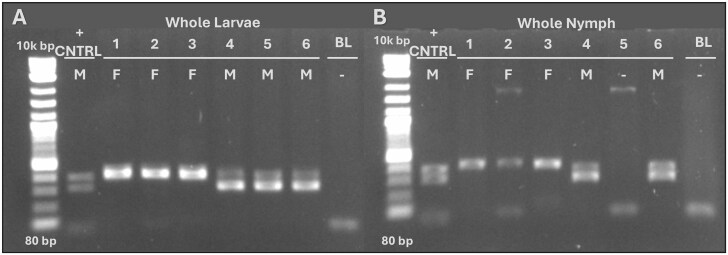
Sex-determination of whole larvae and nymphs. A previously described duplex PCR was used to determine the sex of whole larva and nymph samples (Ronai et al. 2025). A) PCR determined that 3 larvae were female, and 3 larvae were male. B) PCR determined that 3 nymphs were female, 2 nymphs were male, and 1 nymph failed to amplify. From left to right (A and B): Column 1) DNA ladder III, Column 2) positive control (*I. scapularis* adult male) at the 300 and 400 bp position, Columns 3-8) samples, and Column 9) blank (BL) extraction control.

**Fig. 2.**
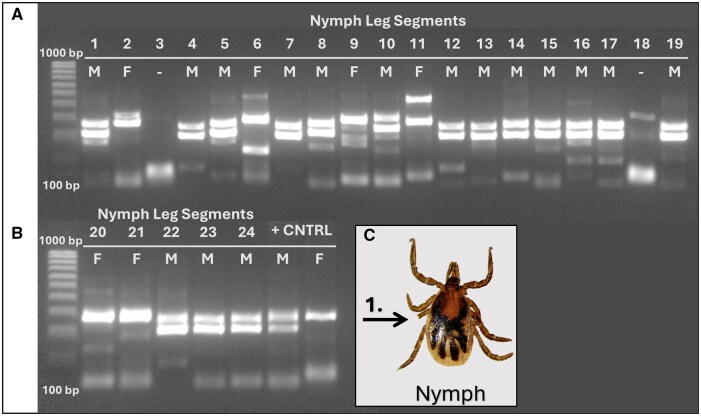
Sex-determination of nymph leg segment. A previously described duplex PCR was used to determine the sex of leg segments of nymph samples (Ronai et al. 2025). PCR determined that 16 samples were male, 6 samples were female, and 2 were inconclusive. A) From left to right: Column 1) DNA ladder II, Column 2-20) Nymph Leg Segments 1 to 19. B) From left to right: Column 1) 100 bp ladder, Column 2-7) Nymph Leg Segments 20 to 24, Column 8-9) Positive *I. scapularis* adult male and female, respectively. C) 24 nymphs had a segment of their third leg removed (1) to be used for molecular sex determination.

The validation and application of a previously described *I. scapularis* sex determining duplex PCR were tested by conducting two experiments. “Experiment 1” consisted of using 6 *I. scapularis* larvae and nymphs each to first determine if whole immature ticks could be used for sex identification (Fig. 1). Immature ticks were placed on double-sided tape and pierced in the center using 31G × 5/16″ insulin syringes (BH Supplies). Immediately after being pierced, ticks were placed in a 200 µl tube containing QuickExtract DNA Extraction Solution (Lucigen), and tick DNA was extracted. “Experiment 2” consisted of first determining the sex of 24 nymphs by extracting DNA from a leg, then allowing nymphs to feed to completion, and validating the sex of the resultant adult tick to the PCR results. Allowing the nymphs to feed and molt into adults provides increased reliability in the methods used to determine sex in the nymph stage. Live nymphs were placed on double-sided tape, and an individual leg was removed up to the patella segment on the third pair of legs using a dissection scope and scalpel (Fig. 2C). The individual leg segment was placed in 200 µl PCR tubes containing DNA QuickExtract DNA Extraction Solution (Lucigen). After leg clipping, the individual nymphs were transferred into 1.5 ml tubes containing a paper towel soaked with 30 μl of MilliQ water. They were allowed to recover from the procedure for a week in an incubator at 20 °C and 95% relative humidity.

### Tick Feeding

Nymphs were fed on the dorsal region of a rabbit purchased from Jackson Laboratory (Bar Harbor, MA, United States). Nymphs were separated into 3 containers based on the putative sex of each nymph determined from the sex-determining PCR (Male, Female, and undetermined). All nymphs were kept at 4 °C for 2 weeks and then moved to 20 °C for 2 d before infesting the rabbit. Capsules were attached to the back of the rabbits similar to that described previously (Reyes et al. 2024). Nymphs were allowed to feed to completion and were placed in individual 1.5 ml tubes containing a damp tissue at 20 °C until molting to adults. Following the molt, ticks were identified as male or female.

### DNA Extraction

DNA from whole immature ticks in experiment 1 and nymph leg segments from experiment 2 were extracted using QuickExtract DNA Extraction Solution (Lucigen). Whole larvae and nymphs were placed in 15 µl of extraction solution while leg segments from nymphs were placed in 10 µl of extraction solution. All samples were vortexed for 5 s, incubated at 65°C for 6 min, vortexed for 15 s, and then incubated at 98 °C for 2 min. Extracted DNA was stored at −20 °C until needed. DNA concentration was measured using a NanoDrop One (ThermoFisher) (Supplementary Table S1) and repeated on a Qubit 2 Fluorometer (Q32866) using a Qubit dsDNA quantification assay (Invitrogen, Q32851) according to the manufacturer’s instructions (Supplementary Table S2).

### PCR and Gel-Electrophoresis

A duplex PCR developed by Ronai et al. (2025) was used to determine the sex of immature ticks in experiments 1 and 2 (Ronai et al. 2025). A single PCR reaction consists of 2 primer pairs, with one amplifying a 326-bp male-specific genomic region (band1), while the other amplifies a 406-bp fragment of the autosomal *40S ribosomal protein S4-like* gene (band2). The sample reaction volume was 20 µl, consisting of 4 µl of 1× Phusion HF Buffer, 0.4 µl of 10 mM dNTP mix (Thermo Scientific), 11.8 µl of nuclease-free water, 0.5 µl (0.25 µM) of the band1 primers, 0.3 µl (0.15 µM) of the band2 primers, 0.2 µl (0.4 U) of Phusion High-Fidelity DNA Polymerase (Thermo Scientific), and 2 µl of template DNA. Amplification settings used were previously described (Ronai et al. 2025). Amplicons were visualized on a 1.25% TBE gel with DNA ladder III (Apex DNA ladder III from 80 bp to 10 kb) (Fig. 1) and a 1.25% TAE gel with DNA ladder II (Apex DNA ladder II from 100 to 1000 bp). The presence of 2 PCR product bands indicated a male sample, while the presence of one band (406 bp) indicated a female sample. *I. scapularis* adult males were used as a positive control, and an extraction control blank was used as a negative control.

## Results

In experiment 1, the duplex PCR was able to amplify target DNA and produce banding patterns that would indicate the ticks were either male or female (Fig. 1). Of the 6 whole larvae, 3 (50%) tested positive for female and 3 (50%) tested positive as male (Fig. 1A). Similarly, of the 6 unfed nymphs, 3 (50%) tested positive as female and 2 (33.3%) tested positive as being male. One (16.7%) unfed nymph sample failed to amplify (Fig. 1B). The size of the bands were consistent with the reported sizes of 406 and 326 bp. Based on the limited sample size, the ability of the duplex PCR to determine the sex of whole immature *I. scapularis* ticks is 91% (10/11).

**Fig. 3.**
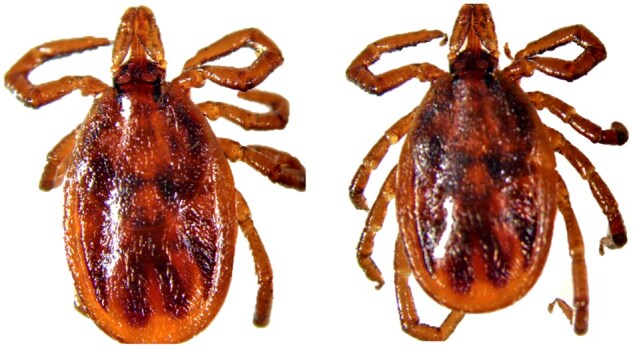
Adult ticks molted from nymphs. Experiment 2: Nymphs were fed on rabbits and allowed to molt into adults. Two of the nymphs molted into adult females which corresponded to their putative sex as determined by PCR.

In experiment 2, the duplex PCR was able to amplify target DNA and produce banding patterns that would indicate the ticks were either male or female for 91.7% (22/24) of the samples (Fig. 2). 66.7% (16/24) of nymphs were determined to be male, and 25% (6/24) were determined to be female. 8.3% (2/24) of the samples were inconclusive. The subset of nymphs that were able to be sexed was then placed on a rabbit to feed in groups that corresponded to their putative sex. Of the 22 nymphs used, only 9.1% (2/22) nymphs fed to completion and molted. These nymphs were putatively characterized as female according to the duplex PCR, and both successfully molted into adult females (Fig. 3).

## Discussion

Based on the limited data, we show that the duplex PCR can determine the sex of *I. scapularis* larvae and nymphs. Although we did not take a portion of tissue from larvae and allow them to become adults, we believe that these results, coupled with the study by Ronai et al. (2025), provide strong evidence to show the validity of the PCR and its application to be used in immature *I. scapularis*. It is likely that most nymphs did not survive the feeding process due to the low humidity conditions in the feeding room, coupled with the loss of the third leg segment of the nymphs, which might have led to hemolymph loss. While there is no study on tick wound healing, an elegant study in locusts showed that the wound repair process does not always occur. From a total of 32 subjects tested, repair (defined as visible deposition of cuticle at the incision site) occurred in only 15 subjects, fewer than 50% (Parle et al. 2016). The researchers suggested that non-repair in these subjects could be due to relatively wide incision, and/or a relative displacement of the cut surfaces, preventing the recovery of a continuous epidermal layer (Parle et al. 2016). Similar recovery of the epidermis and culmination of cuticle secretion might happen at the cut site in tick legs. Using a fine scalpel and straight cut that causes less damage along with the use of wax or silicone at the cut site might result in better survival.

While the data show high reliability of this procedure, we did notice spurious bands and low confidence if we deviated from the published protocol. For instance, when we used a lower DNA concentration or changed the cycle numbers, the data were less reliable (Supplementary Fig. S1), suggesting that the optimized protocol needs to be used. We also noticed that the banding patterns between the adult positive controls and the nymph leg samples were not completely identical (Fig. 2), which we posit could be due to the controls being adults versus using a nymph sample as a control. Future studies could use wax or and allow for the continued survival of ticks through the engorgement and molting process. Since PCR has its limitations, other more sensitive methods, such as qPCR or ddPCR, might provide higher confidence in sexing immature ticks using partial legs of nymphs or larvae.

## Supplementary Material

tjaf138_Supplementary_Data
