# Supplementary material for: Validation of newly synthesized sex-determining PCR using immature Ixodes scapularis (Acari: Ixodidae) ticks
Source: J Med Entomol. 2025 Oct 8;62(6):1622–5. doi: 10.1093/jme/tjaf138 (PMC12616234; doi:10.1093/jme/tjaf138)
Supplement: tjaf138_Supplementary_Data [file tjaf138_supplementary_data.docx]

**Supplementary material:**


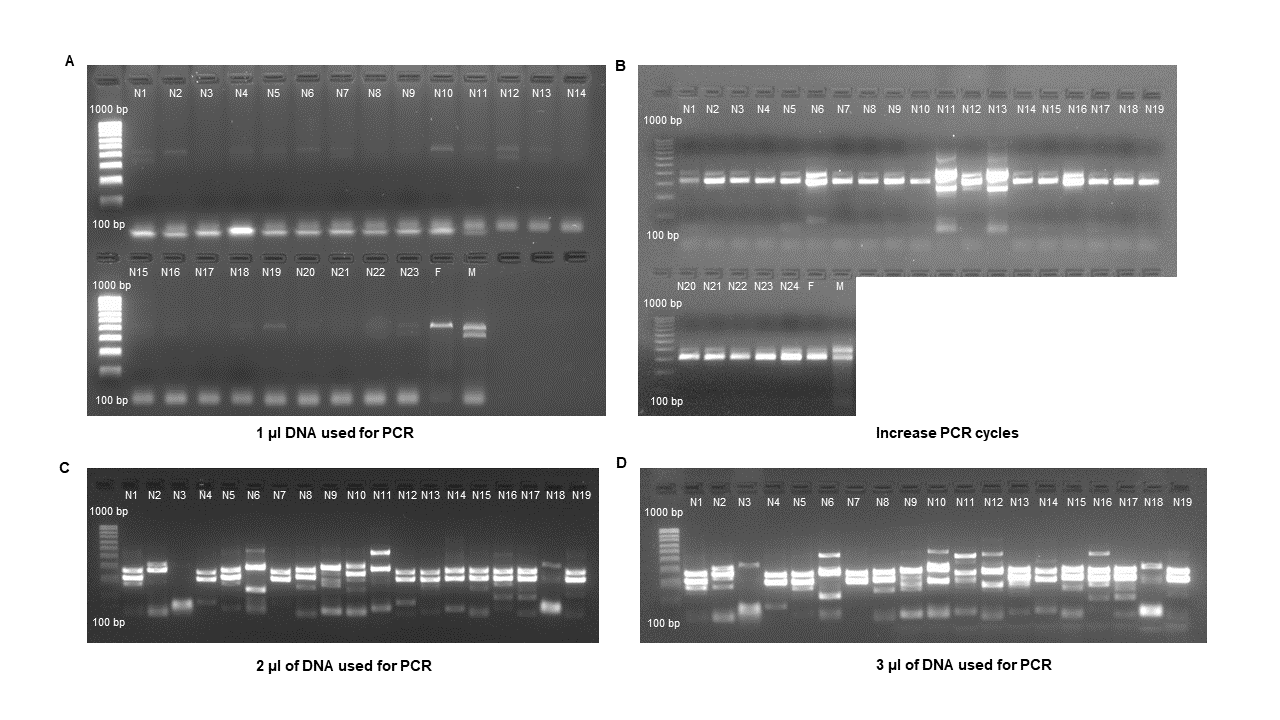


Figure 1: Duplex PCR for DNA from nymph legs with changed reaction: A) With lower DNA concentration (1µl of DNA and 35 amplification cycles), B) With 1µl of DNA and increased number of cycles for PCR (38 cycles), C) With 2µl of DNA and 35 amplification cycle (a portion of gel image from Figure 2), D) 3 µl of DNA and 35 amplification cycles. Samples were run on a 1.25% TAE gel with a 100 bp ladder (DNA ladder II).

Table 1: DNA concentration values of whole larvae/nymphs and nymph leg segments. DNA concentrations were measured on a NanoDrop One.

| **Sample** | **Conc. (ng/µl)** | **A260/280** | **A260/230** |
| --- | --- | --- | --- |
| Whole Larva 1 | 43.4 | 1.17 | 0.70 |
| Whole Larva 2 | 4.9 | 2.15 | 0.08 |
| Whole Larva 3 | 24.8 | 1.63 | 0.26 |
| Whole Larva 4 | 30.3 | 1.31 | 0.31 |
| Whole Larva 5 | 21.6 | 1.67 | 0.27 |
| Whole Larva 6 | 11.8 | 1.67 | 0.22 |
| Whole Nymph 1 | 66.9 | 2.69 | 0.40 |
| Whole Nymph 2 | 12.5 | 1.74 | 0.10 |
| Whole Nymph 3 | 11.0 | 6.26 | 0.18 |
| Whole Nymph 4 | 31.7 | 1.99 | 0.34 |
| Whole Nymph 5 | 6.8 | 2.99 | 0.11 |
| Whole Nymph 6 | 26.4 | 2.13 | 0.33 |
| Blank | 1.5 | 1.71 | 0.03 |
| Nymph Leg 1 | 45.2 | 1.12 | 0.04 |
| Nymph Leg 2 | 34 | 1.25 | 0.03 |
| Nymph Leg 3 | 29.2 | 1.26 | 0.02 |
| Nymph Leg 4 | 25.2 | 1.39 | 0.02 |
| Nymph Leg 5 | 30.8 | 1.33 | 0.03 |
| Nymph Leg 6 | 28 | 1.35 | 0.02 |
| Nymph Leg 7 | 19.7 | 1.55 | 0.02 |
| Nymph Leg 8 | 36.3 | 1.22 | 0.03 |
| Nymph Leg 9 | 56.8 | 0.99 | 0.05 |
| Nymph Leg 10 | -- | -- | -- |
| Nymph Leg 11 | 29.4 | 1.38 | 0.02 |
| Nymph Leg 12 | -- | -- | -- |
| Nymph Leg 13 | 15.2 | 1.84 | 0.02 |
| Nymph Leg 14 | 16.8 | 1.69 | 0.02 |
| Nymph Leg 15 | 20.1 | 1.57 | 0.02 |
| Nymph Leg 16 | 15 | 1.62 | 0.02 |
| Nymph Leg 17 | 15.6 | 1.58 | 0.02 |
| Nymph Leg 18 | 29.3 | 1.22 | 0.02 |
| Nymph Leg 19 | 30.2 | 1.32 | 0.02 |
| Nymph Leg 20 | 13.7 | 1.55 | 0.02 |
| Nymph Leg 21 | 13.1 | 1.66 | 0.02 |
| Nymph Leg 22 | 13.7 | 1.63 | 0.02 |
| Nymph Leg 23 | 11.2 | 1.47 | 0.02 |
| Nymph Leg 24 | 25.2 | 1.42 | 0.02 |

Table 2: DNA concentration values of whole larvae/nymphs and nymph leg segments. DNA concentrations were measured on a Qubit 2 Fluorometer. Nymph leg samples 10 and 12 and whole larvae 6 were unable to be analyzed as no DNA stock remained.

| **Sample** | **Reading (ng/ml)** | **Reading(ng/µl)** | **Dilution factor(200x) correction (ng/µl)** |
| --- | --- | --- | --- |
| Nymph leg 1 | 250 | 0.25 | 50 |
| Nymph leg 2 | 122 | 0.122 | 24.4 |
| Nymph leg 3 | 9.7 | 0.0097 | 1.94 |
| Nymph leg 4 | 169 | 0.169 | 33.8 |
| Nymph leg 5 | 216 | 0.216 | 43.2 |
| Nymph leg 6 | 137 | 0.137 | 27.4 |
| Nymph leg 7 | 239 | 0.239 | 47.8 |
| Nymph leg 8 | 118 | 0.118 | 23.6 |
| Nymph leg 9 | 61.8 | 0.0618 | 12.36 |
| Nymph leg 10 | -- | -- | -- |
| Nymph leg 11 | 127 | 0.127 | 25.4 |
| Nymph leg 12 | -- | -- | -- |
| Nymph leg 13 | 233 | 0.233 | 46.6 |
| Nymph leg 14 | 213 | 0.213 | 42.6 |
| Nymph leg 15 | 111 | 0.111 | 22.2 |
| Nymph leg 16 | 179 | 0.179 | 35.8 |
| Nymph leg 17 | 239 | 0.239 | 47.8 |
| Nymph leg 18 | 12.5 | 0.0125 | 2.5 |
| Nymph leg 19 | 169 | 0.169 | 33.8 |
| Nymph leg 20 | 93.1 | 0.0931 | 18.62 |
| Nymph leg 21 | 106 | 0.106 | 21.2 |
| Nymph leg 22 | 98.6 | 0.0986 | 19.72 |
| Nymph leg 23 | <0.50 | -- | 0 |
| Nymph leg 24 | 1.47 | 0.00147 | 0.294 |
| Whole Nymph 1 | 150 | 0.15 | 30 |
| Whole Nymph 2 | 70.6 | 0.0706 | 14.12 |
| Whole Nymph 3 | 70.6 | 0.0706 | 14.12 |
| Whole Nymph 4 | 56.3 | 0.0563 | 11.26 |
| Whole Nymph 5 | 61.1 | 0.0611 | 12.22 |
| Whole Nymph 6 | 68.9 | 0.0689 | 13.78 |
| Whole Larvae 1 | 13.4 | 0.0134 | 2.68 |
| Whole Larvae 2 | 1.84 | 0.00184 | 0.368 |
| Whole Larvae 3 | 37.3 | 0.0373 | 7.46 |
| Whole Larvae 4 | 27.6 | 0.0276 | 5.52 |
| Whole Larvae 5 | 14.7 | 0.0147 | 2.94 |
| Whole Larvae 6 | -- | -- | -- |
